# Assessing the feasibility of GS FLX Pyrosequencing for sequencing the Atlantic salmon genome

**DOI:** 10.1186/1471-2164-9-404

**Published:** 2008-08-28

**Authors:** Nicole L Quinn, Natasha Levenkova, William Chow, Pascal Bouffard, Keith A Boroevich, James R Knight, Thomas P Jarvie, Krzysztof P Lubieniecki, Brian A Desany, Ben F Koop, Timothy T Harkins, William S Davidson

**Affiliations:** 1 Department of Molecular Biology and Biochemistry, Simon Fraser University, Burnaby, Canada; 2 454 Life Sciences, Branford, USA; 3 Department of Biology, University of Victoria, Victoria, Canada; 4 Roche Applied Science, Indianapolis, USA

## Abstract

**Background:**

With a whole genome duplication event and wealth of biological data, salmonids are excellent model organisms for studying evolutionary processes, fates of duplicated genes and genetic and physiological processes associated with complex behavioral phenotypes. It is surprising therefore, that no salmonid genome has been sequenced. Atlantic salmon (*Salmo salar*) is a good representative salmonid for sequencing given its importance in aquaculture and the genomic resources available. However, the size and complexity of the genome combined with the lack of a sequenced reference genome from a closely related fish makes assembly challenging. Given the cost and time limitations of Sanger sequencing as well as recent improvements to next generation sequencing technologies, we examined the feasibility of using the Genome Sequencer (GS) FLX pyrosequencing system to obtain the sequence of a salmonid genome. Eight pooled BACs belonging to a minimum tiling path covering ~1 Mb of the Atlantic salmon genome were sequenced by GS FLX shotgun and Long Paired End sequencing and compared with a ninth BAC sequenced by Sanger sequencing of a shotgun library.

**Results:**

An initial assembly using only GS FLX shotgun sequences (average read length 248.5 bp) with ~30× coverage allowed gene identification, but was incomplete even when 126 Sanger-generated BAC-end sequences (~0.09× coverage) were incorporated. The addition of paired end sequencing reads (additional ~26× coverage) produced a final assembly comprising 175 contigs assembled into four scaffolds with 171 gaps. Sanger sequencing of the ninth BAC (~10.5× coverage) produced nine contigs and two scaffolds. The number of scaffolds produced by the GS FLX assembly was comparable to Sanger-generated sequencing; however, the number of gaps was much higher in the GS FLX assembly.

**Conclusion:**

These results represent the first use of GS FLX paired end reads for *de novo *sequence assembly. Our data demonstrated that this improved the GS FLX assemblies; however, with respect to *de novo *sequencing of complex genomes, the GS FLX technology is limited to gene mining and establishing a set of ordered sequence contigs. Currently, for a salmonid reference sequence, it appears that a substantial portion of sequencing should be done using Sanger technology.

## Background

The salmonids (salmon, trout and charr) are of considerable environmental, economic and social importance. They contribute to ecosystem health by providing food sources for predators such as bears, eagles, sea lions and whales. As an increasingly popular food choice for humans, salmonid species contribute to local and global economies through fisheries, aquaculture and sport fishing. In addition, they have distinct social importance as they are a traditional food source for indigenous peoples, and play a significant role in their culture and spirituality. Salmonids are also of great scientific interest. The common ancestor of salmonids underwent a whole genome duplication event between 20 and 120 million years ago [1,2]. Thus, the extant salmonid species are considered pseudo-tetraploids whose genomes are in the process of reverting to a stable diploid state. More is known about the biology of salmonids than any other fish group, and in the past 20 years, more than 20,000 reports have been published on their ecology, physiology and genetics. Salmonids, with their genome duplication and wealth of biological data, are excellent model organisms for studying evolutionary processes, fates of duplicated genes and the genetic and physiological processes associated with complex behavioral phenotypes [3]. It is surprising therefore, that no salmonid genome has been sequenced to date.

The Atlantic salmon (*Salmo salar*) is an ideal representative salmonid for genome sequencing given the popularity of this species for aquaculture as well as the extensive genomic resources that are available. The current genomic resources include: a BAC library [4], restriction enzyme fingerprint physical map comprising 223,781 BACs in ~4,300 contigs [5], 207,869 BAC-end sequences that cover ~3.5% of the genome sequence, a linkage map with ~1,600 markers, ~600 of which are integrated with the physical map [6], and > 432,000 ESTs [7,8]. The haploid C-value for Atlantic salmon is estimated to be 3.27 pg [9], or a genome size of approximately 3 × 10^9 ^bp, which is very comparable to the sizes of mammalian genomes. The Atlantic salmon genome is highly repetitive, and at least 14 different DNA transposon families whose members are ~1.5 kb have been described [10]. Although five fish genomes have been sequenced (medaka, *Oryzias latipes*; tiger pufferfish, *Takifugu rubripes; *green spotted pufferfish, *Tetraodon nigriviridis*; zebrafish, *Danio rerio *and stickleback, *Gasterosteus aculeatus*), they represent euteleostei lineages, and often very derived species that have been separated from salmonids for at least 200 million years [11]. The complexity of the Atlantic salmon genome combined with the lack of a closely related guide sequence means that sequencing and assembly will be extremely challenging.

Conventional Sanger sequencing of paired end templates (2–4 kb plasmids, 40 kb fosmids, or ~150 kb BACs) using fluorescent di-deoxy chain terminators and capillary electrophoresis revolutionized the field of genomics (reviewed in [12]). Although this approach remains the gold standard for sequence and assembly quality, limitations with respect to cost, labor-intensiveness and speed, which are largely due to the necessity of generating and arraying cloned shotgun libraries and isolating template DNA for sequencing, have fueled the demand for new approaches to DNA sequencing. In recent years, several novel high-throughput sequencing platforms have entered the market including the SOLiD system by Applied Biosystems [13], the Solexa technology [14], now owned by Illumina, the recently released true Single Molecule Sequencing (tSMS) platform by Helicos [15] and the 454 platform [16], now owned by Roche. Most of these are targeted to the goal of re-sequencing an entire human genome for < $1,000 [17]. This next generation of genome sequencing stands to have major scientific, economic and cultural implications with respect to applications such as personalized medicine, metagenomics and large-scale polymorphism studies on organisms of commercial value whose genomes have already been sequenced. However, the ability of these technologies to sequence the genomes of complex organisms *de novo *remains unknown.

A common feature among the new generation of sequencing procedures is the elimination of the need to clone DNA fragments and the subsequent amplification and purification of DNA templates prior to capillary sequencing. Rather, sequence templates are handled in bulk, and massively parallel sequencing by synthesis or ligation allows the generation of hundreds of thousands to millions of sequences simultaneously.

With respect to *de novo *whole genome sequencing, perhaps the most promising new technology uses a pyrosequencing protocol [18] optimized for solid support and picolitre scale volumes (i.e., pyrosequencing using the 454 system [16]). The 454 pyrosequencing technology [both the Genome Sequencer (GS) 20 and FLX generation systems] has proven very successful for a number of applications such as complete microbial genome sequencing [19] metagenomic and microbial diversity analyses [20,21] ChIP sequencing and epigenetic studies [22,23], genome surveys [24], gene expression profiling [25] and even for sample sequencing fragments of Neanderthal DNA that were extracted from ancient remains [26,27]. Recent accomplishments include its contribution to a high quality draft sequence of the grape genome [28] as well as complete re-sequencing of an individual human genome, for which the assembly was accomplished by mapping 454 reads back to a reference genome [29].

Although several studies comparing 454 pyrosequencing with Sanger sequencing have shown that the per base error rates of the two technologies are similar [27,30], 454 pyrosequencing has limitations. The major concerns have been relatively short read lengths (i.e., as of 2007 an average of 100–200 nt compared to 800–1,000 nt for Sanger sequencing), a lack of a paired end protocol and the accuracy of individual reads for repetitive DNA, particularly in the case of monopolymer repeats [12]. Combined, these factors often make it impossible to span repetitive regions, which therefore collapse into single consensus contigs during sequence assemblies and leave unresolved sequence gaps. These issues have recently been addressed with the release of the GS FLX system as well as the Long Paired End sequencing platform. The GS FLX system provides longer read lengths and lower per-base error rates than the previous systems. In addition, the 454 technology offers the longest read length of any of the next generation sequencing systems currently available. Thus, we chose to evaluate the ability of the 454 technology, as it stands, to sequence a complex genome without the aid of high-coverage Sanger-generated reads.

With respect to *de novo *assembly of a complex genome, the most relevant test to date of the capability of the 454 pyrosequencing technology (GS 20 system) involved sequencing four BACs containing inserts of the barley genome, two of which had previously been sequenced using the traditional Sanger approach [30]. The barley genome is relatively large (5.5 × 10^9 ^bp) and is comprised of more than 80% repetitive DNA, posing a significant challenge for sequencing. Whereas each BAC contained approximately 100 Kb of genomic DNA, the cumulative size of all consensus sequence contigs per BAC did not reach the actual size of the BAC clones for any of the 454-based assemblies. This was largely due to the pooling of repetitive sequences into single contigs. Thus, while the 454 technology proved useful for identifying genes, it was of limited value for producing long contiguous sequence assemblies [30].

Given the significant and ongoing improvements in the 454 technology since the barley BAC analysis, which include longer read lengths and higher sequence accuracy attributable to the release of the GS FLX system, as well as the availability of a paired end protocol, we set out to assess the feasibility of using this technology to sequence the Atlantic salmon genome. Here we report the results of using the GS FLX pyrosequencing system to sequence *de novo *a 1 Mb region of Atlantic salmon DNA covered by a minimum tiling path comprising eight BACs. We discuss the integration of Atlantic salmon genomic resources such as BAC-end sequences as well as assembly techniques and annotation tools given the lack of a closely related guide sequence. We also address the ability of the GS FLX Long Paired End technology to establish the order of sequence contigs and assemble them into large scaffolds. Finally, we compare the GS FLX assemblies with and without the addition of paired end reads to a Sanger-generated assembly of a ninth BAC from the same region of the genome. This is the first application of the GS FLX Long Paired End system for *de novo *assembly of a large region from a complex genome. This study represents the most difficult challenge for 454 pyrosequencing thus far, and the results we present can be used to assess the feasibility of this technology for sequencing the Atlantic salmon genome *de novo*.

## Methods

### Establishment of minimum tiling path and DNA preparation

We initially chose contig 570 of the Atlantic salmon physical map for analysis due to the presence of the microsatellite marker SsaF43NUIG, which is linked to upper temperature tolerance in rainbow trout [31,32] and Arctic charr [33]. Contigs 2469 and 483 were joined to contig 570 using 'chromosome walking'. Specifically, 40-mer oligonucleotide probes were designed from the BAC-end sequences of the outer-most BACs in the contigs, as determined by the contig order predicted by the physical map, beginning with contig 570. The probes were labeled with γ^32^P-ATP using T4 polynucleotide kinase (Invitrogen, Burlington, Ont. Canada) and hybridized to filters containing the Atlantic salmon BAC library [4] (CHORI-214; CHORI, BAC-PAC Resources, Oakland, CA, USA.). Filters were exposed to phosphor screens that were scanned and visualized using ImageQuant™ software, giving an image of the ^32^P-labeled hybridization-positive BACs containing the probe sequence. All hybridization-positive BACs were verified using PCR with the SsaF43NUIG primers [34]. The minimum tiling path across Atlantic salmon contig 483 was established by designing primer sets for sequence tag sites (STSs) in both the SP6 and T7 ends of selected BACs. Using these primers, we screened the BACs that were predicted to overlap with the STS source BAC given the predicted assembly from the Atlantic salmon physical map using PCR, thereby establishing relative BAC orientation and overlap. The minimum tiling path was then established by selecting the minimum number of overlapping BACs required to span the entire contig. We isolated approximately 5 μg of cloned Atlantic salmon BAC DNA from the minimum tiling path BACs using Qiagen's Large Construct kit as per the manufacturer's directions (Qiagen, Mississauga, Ont. Canada). The kit includes an exonuclease digestion step to eliminate *E. coli *genomic DNA.

### 454 shotgun pyrosequencing

The shotgun sequencing protocol using the 454 sequencing system has been described previously [16]. The salmon BAC results presented here were generated on the GS FLX (454 Life Sciences, Branford, CT) whereas the results presented previously [16] were generated on the GS 20 sequencer, the previous generation instrument. The GS FLX instrument is capable of generating 100 million bp of sequence in approximately 250 bp reads in a 7.5 hour run. Additionally, the GS FLX system has a significantly lower error profile than the GS 20 system.

Briefly, to generate the GS FLX shotgun library, the isolated Atlantic salmon BAC DNA was mechanically sheared into fragments, to which process specific A and B adaptors were blunt end ligated. The adaptors contain the amplification and sequencing primers necessary to the GS FLX sequencing process. After adaptor ligation, the fragments were denatured and clonally amplified via emulsion PCR, thereby generating millions of copies of template per bead. The DNA beads were then distributed into picolitre-sized wells on a fibre-optic slide (PicoTiterPlate™), along with a mixture of smaller beads coated with the enzymes required for the pyrosequencing reaction, including the firefly enzyme luciferase. The four DNA nucleotides were then flushed sequentially over the plate. Light signals released upon base incorporation were captured by a CCD camera, and the sequence of bases incorporated per well was stored as a read. DNA extractions were performed at Simon Fraser University (Burnaby, BC, Canada), and library generation and sequencing were performed at 454 Life Sciences (Branford, CT, USA).

### GS FLX Long Paired End DNA library generation and sequencing

GS FLX Long Paired End library generation for 454 sequencing has been described previously [23]. Briefly, DNA was sheared into ~3 kb fragments, EcoRI restriction sites were protected via methylation, and biotinlylated hairpin adaptors (containing an EcoRI site) were ligated to the fragment ends. The fragments were subjected to EcoRI digestion and circularized by ligation of the compatible ends, and subsequently randomly sheared. Biotinlyated linker containing fragments were isolated by streptavidin-affinity purification. These fragments were then subjected to the standard 454 sequencing on the GS FLX system. The paired end reads are recognizable as the known linker (originating from the two hairpin adaptors) surrounded by BAC sequence. When sequenced on the GS FLX, this protocol generates two, ~100 bp tags known to be ~3 kb apart. These paired end reads were used to build the original contigs and to assemble the contigs into scaffolds.

### GS FLX assemblies

A previous version of the Newbler assembler used in performing the assemblies has been described previously [16], and the overall structure and phases of the assembler used here follows the structure described in that paper; however, the algorithms used for the specific phases of assembly have been upgraded. The upgraded Newbler assembler identifies pairwise overlaps between reads, and then uses them to construct multiple alignments of contiguous regions of the dataset. Boundaries where the read-by-read alignments diverge or converge (such as at the boundaries of repeat regions) define breaks in the contig multiple alignments (also called branch points). The resulting data structure consists of a graph, where each node is a contiguous multiple alignment, undirected edges exist between the 5' and 3' ends of the contig nodes, and reads form alignments along paths of the graph. The assembler builds this multiple alignment graph using an adjustable greedy algorithm of taking a 'query' read, finding the pairwise overlaps to it, constructing a multiple alignment of those overlaps, then choosing a subsequent 'query' read from the overlapped reads that are only partially aligned so far (thereby extending the multiple alignment). If any pairwise overlap alignments conflict with the current multiple alignment graph, corrective algorithms use the conflicting alignments to either ignore the new pairwise overlap (if the graph is more consistent) or to correct the constructed multiple alignment (if the new pairwise overlap identifies a misalignment in the graph). These overlaps and multiple alignment algorithms use a combination of nucleotide-space (i.e., the bases of the reads) and flow-space (i.e., the 454 flowgram signal intensities of the reads), where available, to perform the multiple alignment construction.

Following the construction of the multiple alignment graph, a series of 'detangling' algorithms are used to simplify the complex regions of the graph, such as overly collapsed regions shorter than the length of the reads (i.e., parts of reads that happened to be near-identical to each other by chance, and so produced overlaps that collapsed into a single multiple alignment region). The nodes in the resulting graph after detangling are considered to be the 'contigs' by the assembler, and those longer than 500 bp are output as the 'large contigs' of the assembly (those longer than 100 bp are output in the set of 'all contigs').

If paired end reads are included in the data set (either 454 or Sanger paired ends), then an additional scaffolding step is performed after detangling, to create chains of contig nodes using the paired end information. The pairs from each library where both halves of the pair occur in the same contig are used to calculate expected pair distances for the library. The scaffolding algorithm then performs a greedy algorithm of identifying pairs of nodes where at least two paired end reads have their halves aligned at the ends of the pair of nodes, with the correct alignment direction and expected distance from each other. In addition, the set of paired end reads aligned at those two contig ends must support the unambiguous chaining of the two nodes as immediate neighbors in a scaffold, with fewer than 10% of the paired end reads aligning to other contig nodes in the assembly. The chains of contig nodes found by this greedy algorithm are output as the scaffolds of the assembly.

### Gene mining of 454 GS FLX assemblies using syntenic regions

Sequence contigs > 1,000 bp were analyzed using a variety of sequence similarity searches and gene prediction algorithms that have been incorporated into an in-house computational pipeline and database [35]. Sequences entering this pipeline were screened (masked) for repetitive elements using RepeatMasker 3.1.8 [36] and were searched against the NCBI nr (non-redundant) and Atlantic salmon EST [8] databases using BLAST [37]. A GENSCAN gene model prediction algorithm [38] was used to predict introns and exons, and the resulting predictions were searched against the Uniref50 (clustered sets of sequences from UniProt Knowledgebase) database [39]. Finally, a rps-BLAST against the NCBI CDD (Conserved Domain Database; [40]) was conducted to provide additional information with respect to the predicted genes [see additional File 1].

### Use of BAC-end sequences to confirm GS FLX scaffold builds and order

The final scaffold assembly incorporating all data (GS FLX shotgun, paired end and BAC-end reads) was verified by conducting BLAST searches of the 126 BAC-end sequences against the four scaffolds > 10,000 bp and comparing the alignment positions with those predicted by the Atlantic salmon physical map. This method was also used to establish relative scaffold order and to confirm the gene order predicted by the BLAST searches of the 454 shotgun and BAC-end sequence contigs against four published fish genomes.

### Sanger shotgun sequencing, assembly and annotation

The ninth BAC (S0022P24) of the minimum tiling path was sequenced using standard Sanger sequencing of a shotgun library. Briefly, the purified BAC DNA was sheared by sonication and blunt-end repaired. The sonicated DNA was size fractioned by agarose gel electrophoresis and 2–5 kb fragments were purified using the QIAquick Gel Extraction Kit (Qiagen, Mississauga, Ont. Canada). DNA fragments were ligated into pUC19 plasmid that had been digested with SmaI and treated with shrimp-alkaline phosphatase to produce de-phosphorylated blunt ends. The ligation mixture was used to transform supercompetent *E. coli *cells (XL1-Blue; Stratagene, La Jolla, CA. USA). Transformed cells were cultured overnight at 37°C on LB/agar plates supplemented with ampicillin (200 mg/L) and 1,920 (5 × 384 well plates) clones were sent to the Michael Smith Genome Sciences Centre for sequencing. The sequences were analyzed for quality using PHRED [41], assembled using PHRAP [42], and viewed using Consed version 15.0 [43]. The S0022P24 assembly was annotated using the same protocol as the GS FLX assemblies (see above).

## Results and discussion

### Selection of BACs for GS FLX pyrosequencing

**Figure 1:**
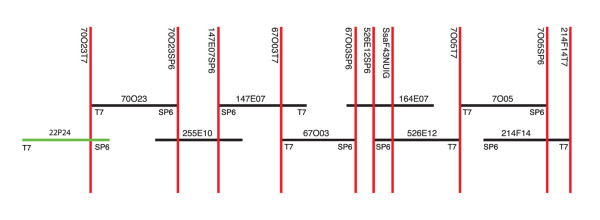
Nine BACs within the minimum tiling path (MTP) of Atlantic salmon contig 483. Using the BAC-end sequences, primers were developed to amplify sequence tag sites (STSs – vertical lines), which were used to design and verify a minimum tiling path across the contig. BAC S0022P24 (green line) was sequenced using traditional Sanger sequencing of a shotgun library and the remaining eight BACs (black lines) were sequenced using the GS FLX platform.

Using chromosome walking, we joined contigs 2469 and 483 to contig 570, and by convention, the new contig was named after the lowest numbered contig within it (i.e., contig 483). Contig 483 contains 195 BACs and includes 126 BAC-end sequences with an average read length of 660 bp. A contig summary can be found in the Atlantic salmon database [6]. Nine BACs were required to span the contig in a minimum tiling path (Fig. 1); eight tiled BACs were selected for GS FLX pyrosequencing and the final (ninth) BAC was sequenced using standard Sanger sequencing of a shotgun library. The estimated length of the minimum tiling path, based on HindIII banding patterns and accounting for overlap between BACs was 1,119,000 bp, with the eight BACs sequenced by GS FLX pyrosequencing accounting for ~950,000 bp. This is probably an underestimate of the true length as doublet and triplet bands may be counted only once.

### GS FLX shotgun assemblies with and without BAC-end sequences

**Figure 2:**
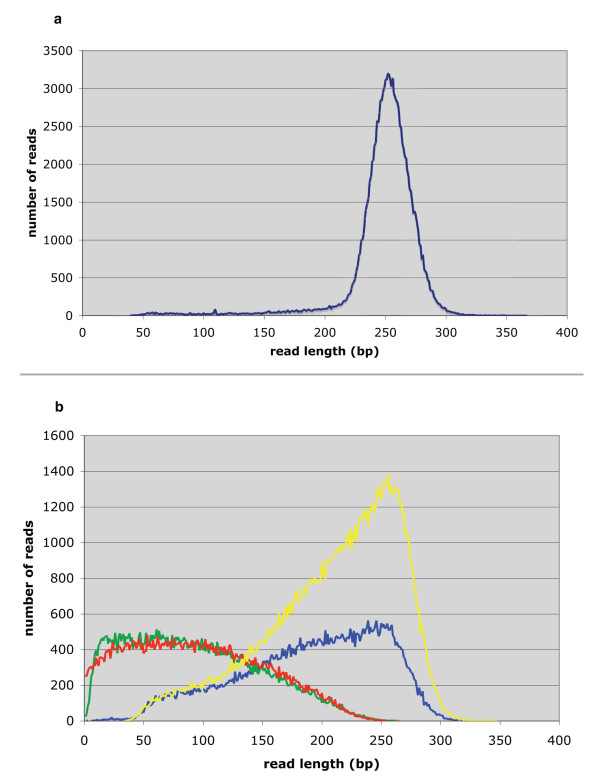
a. Distribution of the read lengths for the GS FLX shotgun sequencing (average 248.5 bp). b. Distribution of read lengths of the GS FLX Long Paired End sequencing. The yellow curve represents the raw reads (average read length 210 bp). These were separated into those containing the linker sequence and those without. The reads containing the linker sequence were separated into two paired end reads, one to the left of the linker (green curve; average read length 93 bp) and those to the right of the linker (red curve; average read length 96 bp). Reads without the linker sequence (blue curve, average read length 191 bp) were added to the assembly as additional shotgun reads.

**Figure 3:**
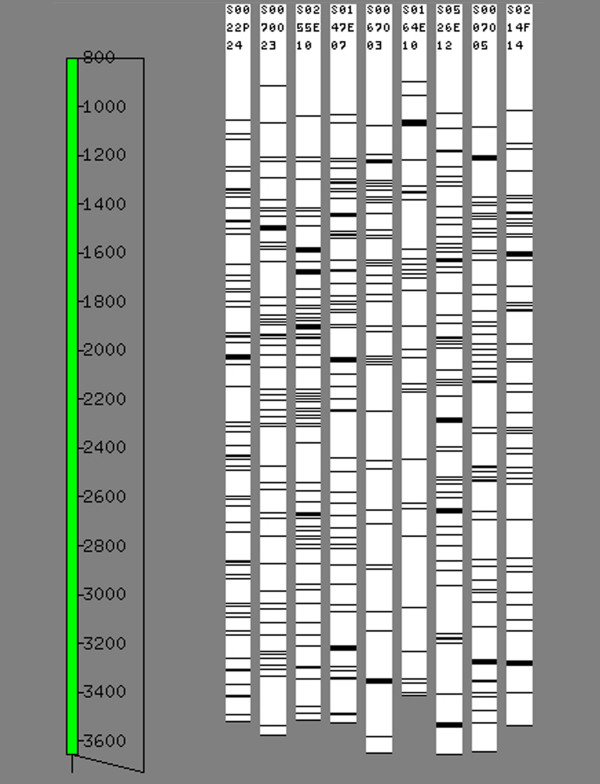
HindIII banding patterns of the nine BACs that comprise the minimum tiling path of contig 483 of the Atlantic salmon physical map. Adjacent lanes share some common bands indicating overlap, whereas lanes separated by more than one lane do not share common bands except when HindIII fragments are of the same size by chance. Scale indicates migration distance. The nine tiled BACs were estimated to span 1,119,000 bp with the eight BACs sequenced by the GS FLX system accounting for approximately 950,000 bp as determined by summing the unique bands in each lane.

We created a GS FLX shotgun library using eight pooled BACs belonging to a minimum tiling path that spanned approximately 1 Mb of the Atlantic salmon genome. The shotgun run produced 141,746 high quality reads with an average read length of 248.5 bp (Fig. 2a). After filtering for vector and *E. coli *sequences, 101,705 reads with a total of 30,549,147 bases were assembled into 803 contigs, 149 of which were > 500 bp and therefore defined as large contigs. Note that this definition of a large contig would include all Sanger-generated reads, which typically range from 500–800 bp. The average contig size was 6,381 bp and the largest contig comprised 34,471 bp. The N50 contig size, defined as the largest contig size at which half of the total size of the contigs is represented by contigs larger than the N50 value, was 11,497 bp (Table 1). The second assembly incorporated an additional 89,095 bp in the form of 126 Sanger-generated BAC-end sequences with an average read length of ~660 bp. This effectively added 126 large contigs to the 149 generated by GS FLX shotgun sequencing. Assembling the GS FLX shotgun data with the BAC-end sequences enabled contig joins, thereby decreasing the number of large contigs to 138 and increasing the N50 contig size to 13,455 bp. The average contig size for the second assembly was 6,827 bp and the largest contig size was 38,211 bp. Both assemblies produced an estimated total length of ~1,080,000 bp not including sequence gaps, which is in agreement with the estimate derived from HindIII fragments (Fig. 3). The GS FLX shotgun sequencing produced ~30× coverage of the region and the BAC-end sequences provided an additional ~0.09× coverage.

**Table 1:** Summary of GS FLX shotgun assemblies

	**SG**	**SG+BE**
Reads assembled	101705	102953
Singleton reads	2795	2870
Large contigs^a ^(> 500 bp)	149	138
Total number of contigs	803	811
Bases in large contigs	950826	942244
Total bases covering region	1088103	1081281
Average contig size (bp)	6381	6827
N50 contig size^b ^(bp)	11497	13455
Largest contig (bp)	34471	38211
> Q40 bases (bp)	947699	939244

GS FLX shotgun assembly alone (SG) and when combined with 126 BAC-end sequences (SG+BE). ^a^Contigs are defined as more than one read joined by overlapping sequence. Large contigs defined as greater than 500 bp. ^b^The N50 contig size is defined as the largest contig size at which half of the total size of the contigs is represented by contigs larger than the N50 value.

### Annotation of GS FLX shotgun contigs > 1,000 bp

**Figure 4:**
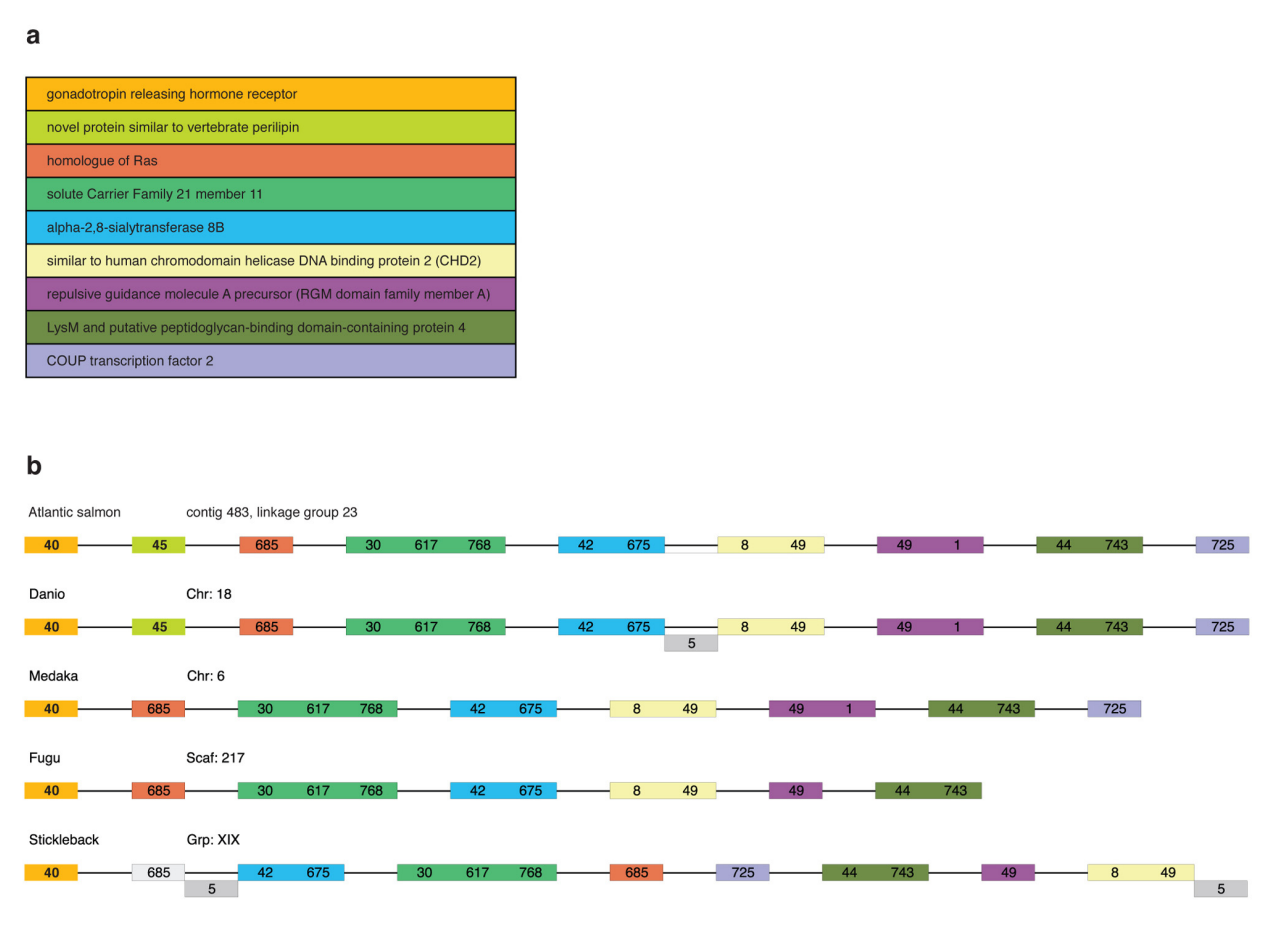
a. Genes identified in the nine BACs using our in-house annotation pipeline . b. Order of the genes within the minimum tiling path. Comparative synteny analysis against the four published fish genomes (medaka, *Oryzias latipes*; tiger pufferfish, *Takifugu rubripes; *green spotted pufferfish, *Tetraodon nigriviridis*; zebrafish, *Danio rerio *and stickleback, *Gasterosteus aculeatus*) enabled the ordering of the gene-containing contigs in the GS FLX assembly of shotgun reads only. This order was confirmed when contigs were assembled into scaffolds with the addition of GS FLX Long Paired End reads. Numbers correspond to contig identity in the Atlantic salmon assemblies; colors coordinate with genes listed in Figure 4a. The grey boxes that correspond to sequence contigs 5 and 685 indicate matches to hypothetical genes. The genes for gonadotropin releasing hormone receptor and the novel protein similar to vertebrate perilipin were found within the Sanger-sequenced BAC and the remaining genes were within the eight BACs sequenced by GS FLX pyrosequencing.

BLAST results for four fish genomes (medaka, *Oryzias latipes*; tiger pufferfish, *Takifugu rubripes; *zebrafish, *Danio rerio *and stickleback, *Gasterosteus aculeatus*) against the large contigs from the GS FLX shotgun and BAC-end sequence assembly revealed hits to seven well annotated genes and one hypothetical gene (Fig. 4a). BLAST results against the *Tetraodon nigriviridis *genome were inconclusive, as most sequence contigs matched to "un_random" sequences (sequence contigs and scaffolds that have not been mapped to any *Tetraodon *chromosome) that collectively spanned over 130 Mb. No genes were identified in any of the fish genomes that were not found in the Atlantic salmon sequence contigs and *vice versa*, indicating conservation of synteny for this genomic region for these four species. Gene order was conserved across three of the four fish species (medaka, zebrafish and the tiger pufferfish), whereas there were two apparent inversions in the stickleback genome relative to the other genomes (Fig. 4b), which may be an artifact of the preliminary, incomplete assembly of the stickleback genome. Using these results and assuming conservation of gene order among teleosts, we could predict the order of 12 gene-containing sequence contigs relative to one another; however, their order with respect to the remaining 126 large contigs could not be established. This confirmed the utility of GS FLX shotgun sequencing for gene discovery and highlighted the difficulty of using this approach alone to assemble the sequence of a complex genome *de novo*.

### Assemblies incorporating GS FLX Long Paired End data

We constructed a GS FLX Paired End library using DNA from the eight tiled BACs to test its ability to improve the shotgun assembly. After trimming for *E. coli *and vector sequences, the GS FLX Long Paired End sequencing produced 149,035 high-quality reads with an average read length of 210 bp (Fig. 2b). Of these, 66,739 contained the linker sequence used to construct the paired end library; therefore, they represented the two paired ends of DNA separated by linker. The average read lengths of the paired ends were 93 and 96 bp for left and right sides of the linker, respectively (Fig. 2b). The remaining reads (i.e., those not containing linker) had an average read length of 191 bp (Fig. 2b) and were used in the assembly as additional shotgun reads. After splitting each linker-containing read into two paired ends and adding the remaining reads, 213,118 usable reads were obtained. When assembled, these produced 310 contigs, 203 of which were assembled into six large scaffolds (i.e., > 10,000 bp) with an N50 scaffold size of 197,327 bp and the largest scaffold was 227,111 bp (Table 2). When combined with the GS FLX shotgun reads, the assembly yielded 289 large contigs, 106 of which were assembled into three large scaffolds with an N50 scaffold size of 361,606 bp and the largest scaffold size was 501,016 bp. Finally, when the 126 BAC-end sequences were incorporated, 286 contigs were produced, 175 of which were assembled into four large scaffolds [GenBank: EU481821] with an N50 and largest scaffold value of 538,994 bp. The GS FLX Long Paired End sequencing provided an additional ~26× coverage of the eight tiled BACs, which, when combined with the GS FLX shotgun data resulted in ~56× coverage of the region. So far, the only published use of the GS FLX Long Paired End technology has been for revealing structural variations in the human genome [23]. The results presented here represent the first use of this technology for *de novo *genome sequence assembly.

**Table 2:** Summary of GS FLX Long Paired End assemblies

	**PE only**	**PE+SG**	**PE+SG+BE**	**S0022P24**
Large contigs^a ^(> 500 bp)	310	289	286	14
Average contig size (bp)	2686	3058	3149	8885
N50 contig size^b ^(bp)	4160	4728	5635	32866
Contigs assembed into scaffolds^c^	203	186	175	9^h^
Total scaffolds	9	3	4	2
Large scaffolds^d ^(> 10 Kb)	6	3	4	2
Average large scaffold size (bp)	96257	299378	226679	112155
Largest scaffold size (bp)	227111	501016	538994	137857
N50 scaffold size^e ^(bp)	197327	361606	538994	137857
Total gaps^f^	194	183	171	8
Maximum gap size (bp)	1,881	2,100	2,131	unknown
Minimum gap size (bp)	4	4	8	unknown
Pair distance average^g ^(bp)	2680	2776	2782	N/A
Pair distance deviation (bp)	670	694	696	N/A
Total bases covering region	958507	1002840	1000926	231017
Depth of coverage	~26×	~56×	~56×	~10.5×

Results for GS FLX Long Range Paired End (PE) assembly alone and when combined with the GS FLX shotgun (SG) data and BAC-end (BE) sequences. ^a^Contigs are defined as more than one read joined by overlapping sequence. Large contigs are greater than 500 bp. ^b^The N50 contig size is defined as the largest contig size at which half of the total size of the contigs is represented by contigs larger than the N50 value. ^c^A scaffold is defined as two or more contigs associated by paired ends. ^d^Large scaffolds are those consisting of more than 10,000 bp among all contigs therein. ^e^The N50 scaffold size is defined as the largest scaffold size at which half of the total size of the scaffolds is represented by scaffolds larger than the N50 value. ^f^Gaps represent unsequenced regions between two contigs known to be adjacent due to associated paired ends. ^g^Average pair distance is the average distance between two sections of BAC DNA separated by linker sequence. ^h^Assembly based on large contigs (> 500 bp) consisting of ≥3 reads each.

The combination of GS FLX shotgun and Long Paired End reads provided approximately 56× coverage of the 1 Mb region of the salmon genome. We speculate that this represents extensive over-coverage and that similar results could be obtained using fewer reads and less coverage of the region. However, further studies that examine various combinations of coverage from shotgun and paired end libraries are necessary to test this hypothesis and to determine the optimal combination of the two GS FLX read types for genome assembly.

### Use of BAC-end sequences and minimum tiling path to confirm assembly and order of scaffolds

**Figure 5:**
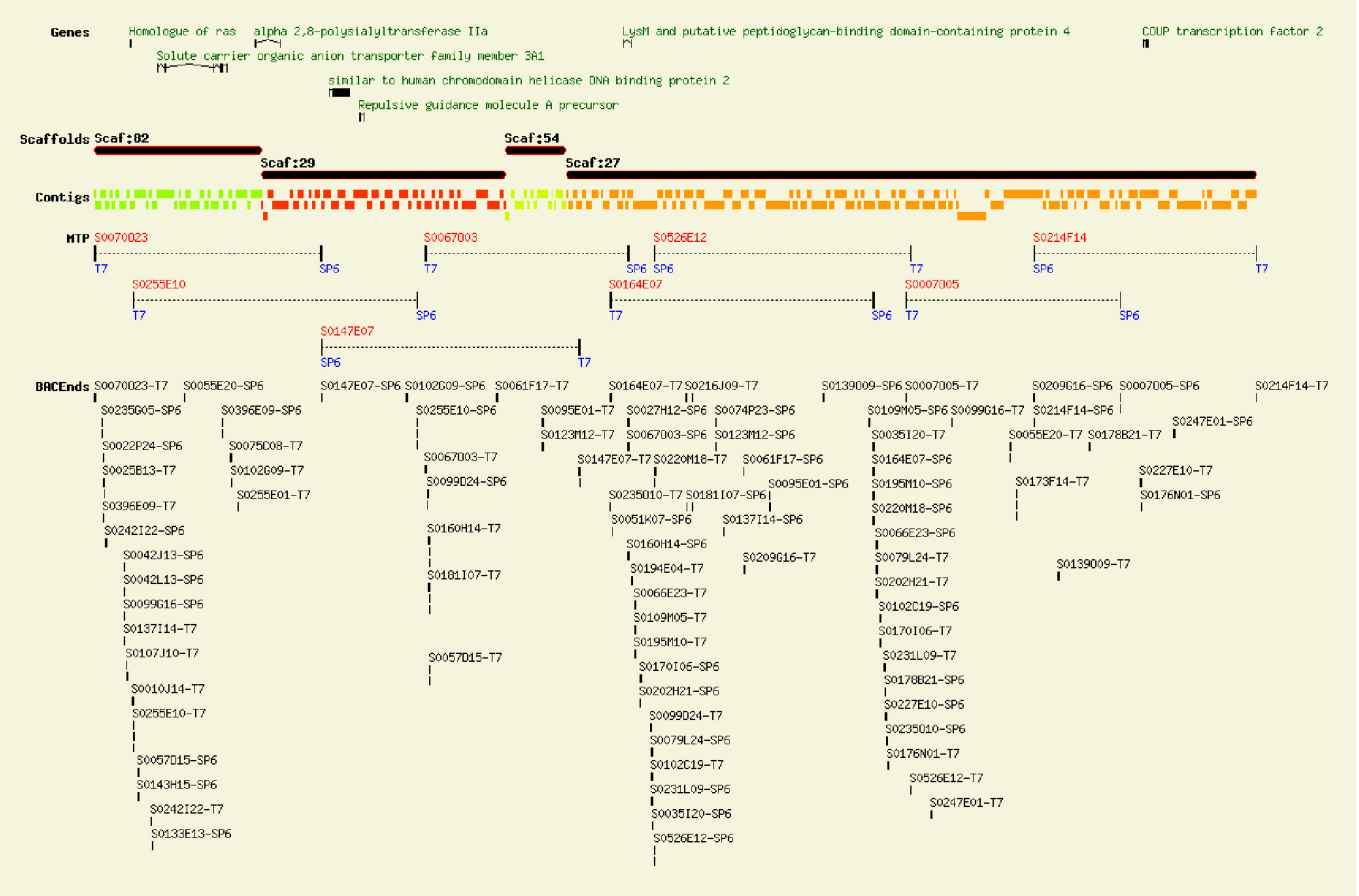
Summary of the 1 Mb sequenced region for the final assembly incorporating the GS FLX shotgun and paired end data with the 126 BAC-end sequences. This figure summarizes all genes identified within the 1 Mb region and their position, the arrangement of the large scaffolds (order and orientation) as confirmed by the BAC-end sequences, the sequence contigs aligned against the scaffolds, the eight BACs of the minimum tiling path (MTP) including established overlap, and the BAC-end sequences within the region in the order predicted by the Atlantic salmon physical map.

The accuracy of the final scaffold assembly was verified by conducting a BLAST search of the 126 BAC-end sequences against the scaffold builds. This also established the order of the four scaffolds relative to one another and confirmed that the aligned sequences followed the order predicted by the minimum tiling path of the eight BACs. These results provided further support for conservation of synteny and gene order of the seven genes in the genomes of Atlantic salmon, medaka, zebrafish and tiger pufferfish. Fig. 5 provides a visual summary of the data, including the minimum tiling path, sequence contigs, scaffolds, predicted genes and BAC-end sequences in the 1 Mb region.

### Assembly and annotation of the ninth BAC

Sanger sequencing of the shotgun library of the ninth BAC (S0022P24) in the minimum tiling path produced 3,524 confirmed reads and an average confirmed read length of 693.3 bp. PHRAP defines a confirmed read as verification of a read by another read with different chemistry or by an opposite-strand read [44]. This produced a ~10.5× depth of coverage given the estimated BAC size of 231,979 bp. The confirmed reads were assembled into 20 contigs with an average contig size of 8,885 bp and an N50 contig size of 32,866 bp; 14 contigs were defined as large contigs (i.e., > 500 bp). Nine large contigs consisting of three or more reads were assembled into two large scaffolds based on corresponding paired end reads from cloned inserts [GenBank: EU873552]. The average and N50 scaffold sizes were 112,155 and 137,857 bp, respectively. The two scaffolds were oriented relative to one another based on the locations of the T7 and SP6 BAC-end sequences.

The Sanger assembly produced a much larger average contig size and N50 contig size than any of the GS FLX assemblies (i.e., with and without paired end and BAC-end sequence reads), which corresponds to fewer contigs produced. This is likely because of the larger average read length of the Sanger sequences. The Sanger assembly produced two scaffolds with eight gaps for a ~230,000 bp region, whereas the final GS FLX assembly produced four scaffolds with 171 gaps for a ~1 MB region. Thus, with respect to the ability to establish the order and orientation of sequence contigs relative to one another, the GS FLX assembly was comparable to a Sanger-based assembly. This, however, was offset by the numerous gaps between contigs within the GS FLX assembly.

Sequence annotation using our in-house pipeline (described above) revealed hits to two genes: gonadotropin-releasing hormone receptor type I and a novel protein similar to vertebrate perilipin (Fig. 4a), with the latter located next to the final gene in the BACs sequenced by GS FLX. When the region was compared with regions that were previously identified as being syntenic with other sequenced fish genomes, only that of the zebrafish (*Danio rerio*) contained both genes. The remaining genomes (medaka, *Oryzias latipes*; tiger pufferfish, *Takifugu rubripes*; and stickleback, *Gasterosteus aculeatus*) only contained the gonadotropin-releasing hormone receptor type I gene with no evidence of the novel protein similar to perilipin or any other genes (Fig. 4b).

### Nature of gaps in GS FLX assembly

**Figure 6:**
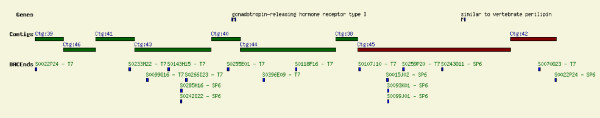
Summary of the Sanger-sequenced BAC (S0022P24). The two genes within the ~200,000 bp region are indicated as well as the nine sequence contigs and two scaffolds (indicated by red and green contigs). The relative orientation of these scaffolds was determined knowing the SP6 and T7 BAC-end sequences. The BAC-end sequences within the region are indicated in the order predicted by the Atlantic salmon physical map. Note that this BAC overlaps with the remainder of the MTP (i.e., that sequenced by GS FLX) at the 70O23-T7 BAC-end.

A major concern is that 171 gaps remain between the GS FLX-sequenced contigs within the four final scaffolds. Given that GS 20, and by extension GS FLX, pyrosequencing is known to provide good coverage of genic regions [24], these gaps likely represent repeat regions rather than missed genes. This was supported by synteny analysis, which indicated that the initial assembly covered all genes present within this region in sequenced fish genomes, and by conducting a BLAST search of gap ends, which revealed that many of the gaps bordered known salmonid repetitive elements [10]. A comparison of the overlapping region between the BAC sequenced by the Sanger method and the corresponding region sequenced by GS FLX pyrosequencing (i.e., the region between the BAC-ends S0070O23-T7 and S0022P24-SP6 in Fig. 6), identified two gaps of 893 and 151 bp in the GS FLX assembly. These regions of the Sanger assembly were completely masked by the salmonid-specific repeat masker [45], thus verifying that the GS FLX technology has difficulty with repetitive regions.

## Conclusion

With 30–40% repetitive content and its pseudo-tetraploid nature due to a whole genome duplication event [2], the Atlantic salmon genome poses a significant challenge for sequencing. To date, the strategies to sequence complex vertebrate genomes have been Sanger sequencing of whole genome shotgun libraries (e.g., dog genome [46]), the generation of a library of cloned inserts such as BACs, followed by a 'map-first, sequence second' approach (e.g., pig genome [47]), or a combination of whole genome shotgun sequencing and pooled BAC sequencing [48]. These strategies are dependent on the minimal ability to sequence and assemble a full BAC insert. However, to date, this has proved unsuccessful with respect to complex genomes with any technique other than Sanger sequencing of a subcloned shotgun library [30].

The purpose of this study was to assess the feasibility of GS FLX pyrosequencing for *de novo *assembly of the Atlantic salmon genome given recent advances in read length and the availability of GS FLX Long Paired End technology. We demonstrated that without the inclusion of GS FLX Paired End reads, the GS FLX shotgun technology alone was substantially inferior to Sanger sequencing given the size and number of contigs produced and the inability to establish the relative order and orientation of the contigs. However, the addition of GS FLX Paired End reads vastly improved the capability of 454 pyrosequencing by enabling the assembly of contigs into large scaffolds. Indeed, in terms of the number of scaffolds produced, the GS FLX assembly that included the combined shotgun and paired end reads was comparable to the Sanger assembly. Moreover, the order of the GS FLX scaffolds could be established from information from BAC-end sequences and the Atlantic salmon physical map. However, numerous gaps remained within the scaffolds, which is undesirable when a complete or reference genome sequence is one of the goals. Currently, if the Atlantic salmon genome is to provide a reference sequence for all salmonids, then a substantial proportion of the sequencing will have to be carried out using Sanger technology.

## Authors' contributions

NLQ, PB, TPJ, BD, JK, TTH, BFK and WSD conceived the project. NLQ established the minimum tiling path and prepared the DNA. PB was responsible for GS FLX pyrosequencing. NL, WC, KAB, JK, KPL and BD performed bioinformatics. NLQ, NL, WC, PB, JK, KAB, KPL and WSD analyzed and interpreted the data. NLQ, TTH and WSD prepared the manuscript.

## Supplementary Material

Additional file 1 Summary of information used for sequence annotation. Species, Ensembl names, assembly release date, Genebuild and database versions for all genome sequences used for comparative synteny analyses of the GS FLX shotgun + BAC-end sequence-generated contigs. Click here for file
