# Supplementary figures and images for: Assessing the feasibility of GS FLX Pyrosequencing for sequencing the Atlantic salmon genome
Source: BMC Genomics. 2008 Aug 28;9:404. doi: 10.1186/1471-2164-9-404 (PMC2532694; doi:10.1186/1471-2164-9-404)

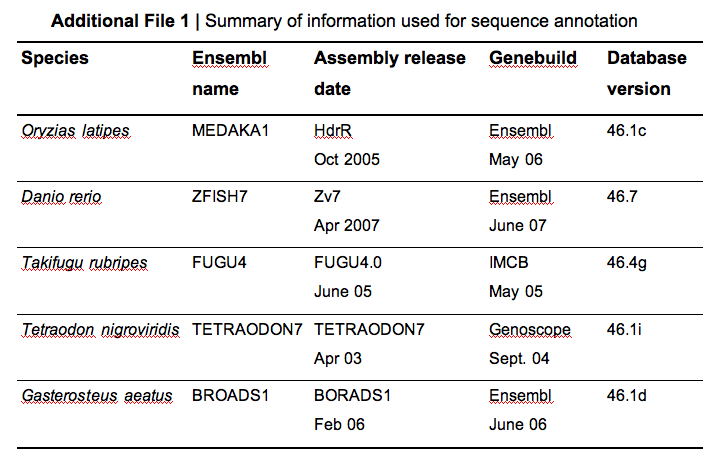

Supplement: Additional file 1 — Summary of information used for sequence annotation. Species, Ensembl names, assembly release date, Genebuild and database versions for all genome sequences used for comparative synteny analyses of the GS FLX shotgun + BAC-end sequence-generated contigs. [file 1471-2164-9-404-S1.jpeg]
